# DelInsCaller: An Efficient Algorithm for Identifying Delins and Estimating Haplotypes from Long Reads with High Level of Sequencing Errors

**DOI:** 10.3390/genes14010004

**Published:** 2022-12-20

**Authors:** Shenjie Wang, Xuanping Zhang, Geng Qiang, Jiayin Wang

**Affiliations:** 1 School of Computer Science and Technology, Xi’an Jiaotong University, Xi’an 710049, China; 2 Shaanxi Engineering Research Center of Medical and Health Big Data, Xi’an Jiaotong University, Xi’an 710049, China

**Keywords:** sequencing data analysis, variant calling, variant phasing, delins, complex indel

## Abstract

Delins, as known as complex indel, is a combined genomic structural variation formed by deleting and inserting DNA fragments at a common genomic location. Recent studies emphasized the importance of delins in cancer diagnosis and treatment. Although the long reads from PacBio CLR sequencing significantly facilitate delins calling, the existing approaches still encounter computational challenges from the high level of sequencing errors, and often introduce errors in genotyping and phasing delins. In this paper, we propose an efficient algorithmic pipeline, named delInsCaller, to identify delins on haplotype resolution from the PacBio CLR sequencing data. delInsCaller design a fault-tolerant method by calculating a variation density score, which helps to locate the candidate mutational regions under a high-level of sequencing errors. It adopts a base association-based contig splicing method, which facilitates contig splicing in the presence of false-positive interference. We conducted a series of experiments on simulated datasets, and the results showed that delInsCaller outperformed several state-of-the-art approaches, e.g., SVseq3, across a wide range of parameter settings, such as read depth, sequencing error rates, etc. delInsCaller often obtained higher f-measures than other approaches; specifically, it was able to maintain advantages at ~15% sequencing errors. delInsCaller was able to significantly improve the N50 values with almost no loss of haplotype accuracy compared with the existing approach as well.

## 1. Introduction

Genomic structural variations (SVs) generally include deletions, tandem duplications, insertions, inversions, translocations, and their combinations [1,2]. Delins, as known as complex indel, is one of those combinations formed by simultaneously deleting and inserting DNA fragments of different sizes at a common genomic location [3,4]. Delins are widely reported in different researches, some of which are considered functional or susceptible [5,6]. For example, some delins may be inherited which are identified and validated from familial research [7,8]. While the germline delins are observed in populations, some somatic delins are reported potentially druggable [9,10]. Thus, identifying delins from sequencing data is a necessary task for data analysis.

Several approaches, such as Pindel-C [3], SV-Bay [11], INDELseek [12], have been developed for identifying delins from the second generation sequencing data. However, the relatively short read length limits the alignment and assembly, thus affecting the performance on variant calling, genotyping and phasing. With the rapid development of sequencing technologies, long read enables high-confidence mapping across a greater percentage of the genome [13,14]. The PacBio SMRT sequencing technology, as a representative of the third generation sequencing technology, has been attracting more and more attention since its commercial release in 2010 [15]. With long reads, structural variations that are previously undetectable in the second generation data can be accurately detected [16]. Currently, the detection algorithm of delins for long reads is SVseq3 [17]. SVseq3 identifies a series of suspicious regions from the aligned reads mapped by BLASR [18]. Although the SVseq3 can process the third generation sequencing data with better f-measure, it can only tolerate up to 3% of sequencing errors. However, PacBio SMRT sequencing technology produces two types of reads: (i) continuous long reads (CLR) (long reads with high error rates) and (ii) circular consensus sequencing (CCS) reads (short reads with low error rates) [19]. For CLR reads, the sequencing error rate is up to 15%, which would result in a quite mix of true and false positive variations. When SVseq3 is processing such data, it always fails due to its inability to distinguish between true and false positive variations.

In addition to variant calling, estimating haplotypes is another important task [20]. For example, recent studies suggest that the patients with the same level of mutation loads may lead to different clinical manifestation when the loads on haplotypes are significantly different. Benefiting from the CLR reads with the length ranging from 15,000 to 40,000 bps, we are empowered to estimate each haplotype by identifying these variations that are co-located on the same read/contig. Currently, the tools for haplotype estimation based on variant detection results include hapcut2, whatshap, etc. However, these tools are prone to incorrect splicing due to the false positives introduced by high sequencing errors.

To summarize above, the existing methods often encounter the following two computational problems. delins is difficult to detect accurately due to the interference of sequencing errors. In addition, the false positives introduced by sequencing errors often mislead contig splicing. But we do need the read length advantage of CLR sequencing in some scenarios. Motivated by this, we propose an efficient algorithmic pipeline, named delInsCaller, to identify the delins on haplotype resolution from the PacBio CLR sequencing data with high sequencing errors. delInsCaller design a fault-tolerant method by calculating a variation density score, which helps to locate the candidate mutational regions under a high-level of sequencing errors. It adopts a base association-based contig splicing method, which facilitates contig splicing in the presence of false-positive interference. The experiments showed that delinsCaller was effective in identifying delins on haplotype resolution, and outperformed several state-of-the-art approaches.

## 2. Materials and Methods

The proposed analysis pipeline consists of the following components. First, it determines the approximate region of a variant by calculating a series of variation density fractions. Then, the candidate variant is classified by multiple machine learning models. A local re-alignment component then locates the exact breakpoints according to the soft clip alignments and estimates the possible source of the inserted fragments. Finally, it estimates the haplotypes for the identified variants.

### 2.1. Identifying Regions of Variations

We extract variant signatures from the SAM file to identify the genomic structural variation region. Due to the sequencing errors, these variant signatures imply not only the true variants, but the false positives as well. Previous studies report that the sequencing errors of CLR reads are almost uniformly distributed, dominated by the fake insertions and deletions [21]. Therefore, the unmatched base density on the reads mapped to a normal region usually locates in a low level. In contrast, the reads mapped to the region with variations may have a relatively higher density. According to this experience, we propose to calculate the variation fraction, which measures the unmatched base density in different regions.

The core idea is as follows: (1) we define a value called variation aggregation degree, which measures the mutation load on a specific region. According to the error model, for any site, we are not sure whether it is a variant or not, but the surrounding sites provide more information. When there are many unmatched sites around, it indicates that this region has a higher variation aggregation degree, and then it has higher probability of being a variation region. (2) In addition, we borrow the idea that the loci in close proximity may have a stronger linkage. The linkage strength sometimes decays exponentially as the physical distance becomes larger [22]. Thus, the mapping status of the surrounding sites are assigned different weights according to the distance from the central site. The closer the site locates to the central, the higher weight is assigned. Now, we calculate a variation score for each site.

Specifically, when traversing sites, we set a sliding window with the size of windows_len and calculate the proportion of unmatched bases at any site k in the window, that is, the proportion of the number of reads harboring unmatched bases to the number of reads covered this site. The formula is as follow:

(1) $$p_{c}\left(k\right)=\frac{u}{d}$$

where c represents the central site of this window, u represents the number of reads containing unmatched bases, and d represents the total number of reads covering this site. For any site k in the window, we define the weight coefficient to measure its influence on the central site. Two conditions are satisfied: (1) The value of k is inversely proportional to the distance between k and c; (2) When the unmatched rate of all sites in the window (c − windows_len, c + windows_len) is 1, the variation score is defined as 1. The formulas are as follows:

(2) $$w_{c}\left(k\right)\propto \frac{1}{l_{k-c}}$$

(3) $$\overset{c+\mathrm{windows}\_\mathrm{len}}{\underset{k=c-\mathrm{windows}\_\mathrm{len}}{\sum }}w_{c}\left(k\right)=1$$

where $l_{k-c}$ represents the distance between k and c. Since standard normal distribution meets the formula $\int_{-3}^{3}f\left(x\right)dx$ = 0.9974 (approximate to 1), we simply adopt the standard normal distribution as the weight coefficient function here. The calculation of the weight coefficient $w_{c}\left(k\right)$ is:

(4) $$\left\{\begin{array}{c} f\left(x\right)=\frac{1}{\sqrt{2\pi }}e^{\left(-\frac{x^{2}}{2}\right)}\\ w_{c}\left(k\right)=\frac{3}{\mathrm{windows}\_\mathrm{len}}f\left(\frac{3\left|k-c\right|}{\mathrm{windows}\_\mathrm{len}}\right) \end{array}\right.$$

Now, we obtain the calculation formula of the variation score as:

(5) $$Score_{c}=\overset{c+\mathrm{windows}\_\mathrm{len}}{\underset{k=c-w\mathrm{indows}\_\mathrm{len}}{\sum }}w_{c}\left(k\right)p_{c}\left(k\right)$$

**Figure 1:**
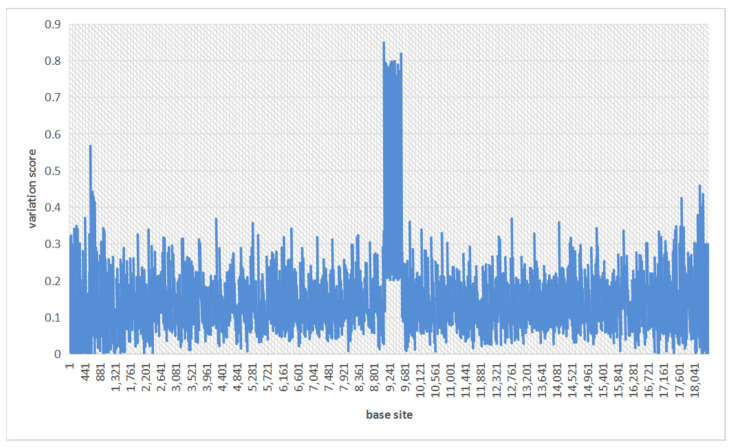
The variation scores for each site on a region under a 15% sequencing error rate.

This variation score has the following features: (1) For any site, the value of variation score is not only related to itself, but related to the surrounding sites as well; (2) If all the sites in the window are mutations, the variation score is approximately equal to 1; while if all the sites are matched to the reference, the variation score is equal to 0; (3) The sites around site c have different effects on the variation score, which depends on the distance to site c. Figure 1 shows an example of the variation scores on a region, when the sequencing error rate reaches 15%. 

According to Figure 1, the portion where the variation score is significantly higher than the surrounding area is the approximate region where we have tentatively identified possible variations. To refine the range of the variation interval, we traverse site i in turn and smooth the curve to eliminate some noise:

(6) $$smoothScore_{i}=\frac{1}{2*\mathrm{windows}\_\mathrm{len}+1}\overset{i+win}{\underset{j=i-win}{\sum }}Score_{i}$$

**Figure 2:**
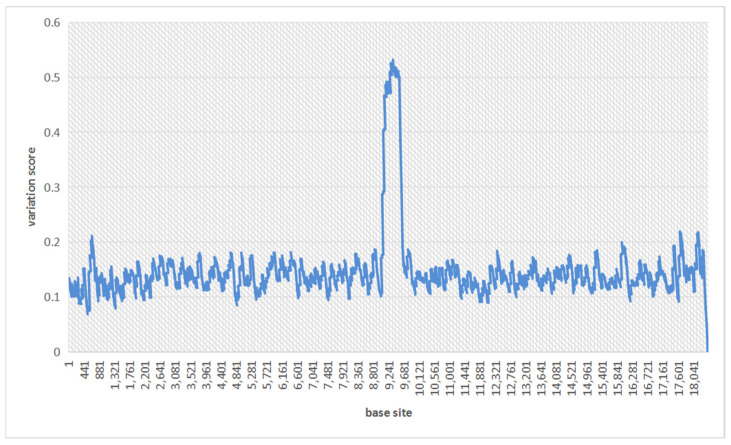
The smoothed variation scores on the same region under a 15% sequencing error rate.

Figure 2 shows the smoothed variation score graph. Obviously, we are able to obtain a clear candidate interval of a structural variation.

We further set a threshold T for variation scores to determine whether a region, after smoothing, is a candidate region or not. For interval $\left(l\;,r\right)$, when it satisfies:

(7) $$\left\{\begin{array}{c} smoothScore_{i}>T\;\;\;\;\;\;\;\;\;\;\;\;\;\;\;\;\;\;\;\;\;\;\;\;\;\;\;\;\;\;\;\;\;\;\;\;\;\;\;\;\;\;\;\;\;\;\;\;\;\;\;\;\;\\ smoothScore_{l-1}<T\;\;\;\;\;\;\;\;\;\;\;\;\;\;\;\;\;\;\;\;\;\;\;\;\;\;\;\;\;\;\;\;\;i\in \left(l\;,r\right)\\ smoothScore_{r+1}<T\;\;\;\;\;\;\;\;\;\;\;\;\;\;\;\;\;\;\;\;\;\;\;\;\;\;\;\;\;\;\;\;\;\;\;\;\;\;\;\;\;\;\;\;\;\;\;\;\; \end{array}\right.$$

We select $\left(l\;,r\right)$ as the candidate region for a structural variation. According to this pipeline, the approximate ranges of variations can be located quickly, in the case of high sequencing errors.

### 2.2. Classification of Variations

When we have the candidate regions, we have to further identify the type of structural variations. The variation could be delins, insertion, deletion, or some other type of structural variation. Due to the sequencing errors, we cannot use the hard-filters to identify the types of variations, because the differences among these variations in the space of features are confused. Thus, we trained multiple SVMs to implement a multiple classifications. An SVM classifier is trained between each of the two variation types. For m types of variations, a total of $\frac{m\left(m-1\right)}{2}$ SVM classifiers were trained. We take the variation type with the largest count according to the results of all classifiers.

To obtain a better classification performance, we study the features of various regions harboring different variations. Since the insertions and deletions often have higher insertion rates and deletion rates, respectively, while delins have higher transition ratios, these features are selected as a group. Since the unmatched sites in insertions and deletions are generally continuous, while the continuity of the unmatched sites in delins is poor, we select the maximum ratio of consecutive unmatched bases and the corresponding ratios as features.

### 2.3. Locating the Start and End Sites of the Variation

**Figure 3:**
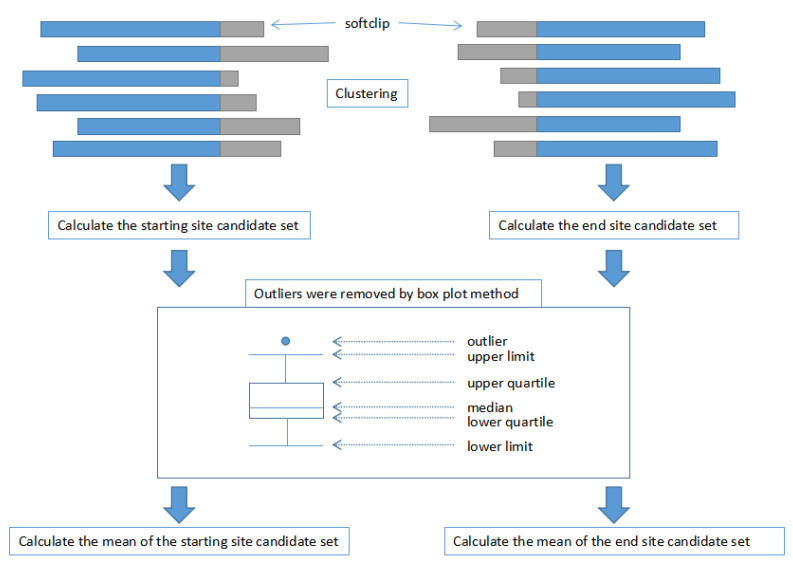
The flow chart of the key steps for locating the start and end sites of a variation.

In this step, we are trying to locate the specific start and end sites of a variation. First, we locate the start site of a variation: (1) Cluster all reads with soft clips at the right end and the length of the soft clip greater than 50 bp in the variation interval defined in the first step. (2) Calculate the starting site of each read in the cluster according to the cigar value of each read to obtain a candidate set of the starting site. (3) Outliers in the candidate set are eliminated by the box diagram method. (4) The mean value of the starting site candidate set is calculated as the starting site for the variation. Next, we locate the end site of the variation: (1) Cluster all reads with soft clips at the left end and the length of the soft clip greater than 50 bp in the variation interval defined in the first step. (2) Calculate the ending site of each read in the cluster according to the cigar value of each read to obtain a candidate set of the ending site. (3) Outliers in the candidate set are eliminated by the box diagram method. (4) The mean value of the ending site candidate set is calculated as the ending site for the variation. A simple flow chart explains this algorithm is shown in Figure 3.

### 2.4. Finding the Source of the Inserted Fragment

**Figure 4:**
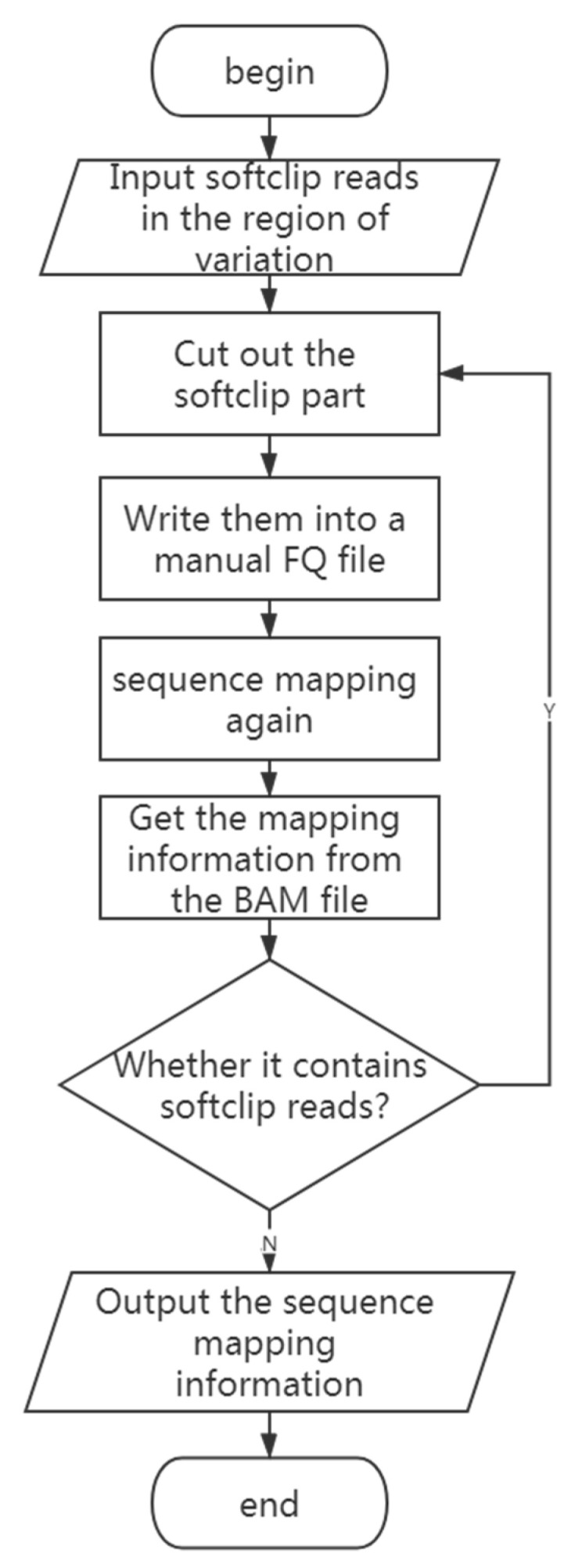
The flow chart for finding the sources of the inserted fragment.

After obtaining the start and end location of the mutation and the type of the mutation, the algorithm traces the source of inserted fragments in the delins variant and insertion variant. The approximate steps, shown in Figure 4, include: (1) The reads containing soft clips in the interval of the variation are cut to obtain the base sequence of the soft clip part, and the base sequence is written into a new FQ file as artificial reads. (2) The new FQ file is matched back to the reference genome, and the BAM file is generated. (3) The longest continuous matching fragment is selected from the sequence mapping results to determine its mapping site. If soft clips are still included, back to step (1). Otherwise, the algorithm is terminated.

### 2.5. Estimating Haplotypes

**Figure 5:**
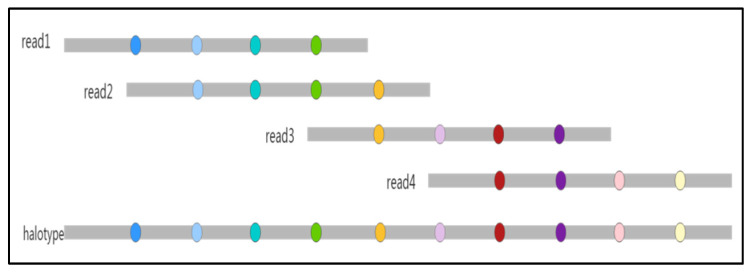
The schematic of estimating a haplotype. The different colored circles represent different variants.

We have obtained the identification results of insertions, deletions, and delins through the previous steps. To further estimate haplotype, we introduce information about single point variation in the sequenced samples (the information about single point variation can be obtained with software such as deepvariant, clairvoyante, NanoCaller [23], and so on) and split the variation into two sets by splicing reads containing the same variations (the HapCUT2 can extract variation information from VCF file and splice them into contigs). For intervals that could not be spliced, we estimate their correlations using linkage disequilibrium value. The formula is as follows:

(8) $$L=\overset{m}{\underset{i=1}{\sum }}\overset{n}{\underset{j=1}{\sum }}lq*p$$

where L represents the correlation of two contigs, *lq* represents the linkage strength of two variants, *p* represents the frequency of variants, and *m* and *n* represent the number of variants in the two contigs, respectively. For two contigs that need to be spliced, first, we calculate the correlation strength of the first polymorphic site base in the posterior contig based on the bases of each polymorphic site in the anterior contig. Then, the same operation is repeated for each posterior base until the correlation calculation is completed for each polymorphic site of the posterior base, and finally, the correlation strengths of the two contigs are accumulated. We select the posterior contig with the higher correlation strength to splice with the anterior contig. The splicing schematic is shown in Figure 5. 

**Figure 6:**
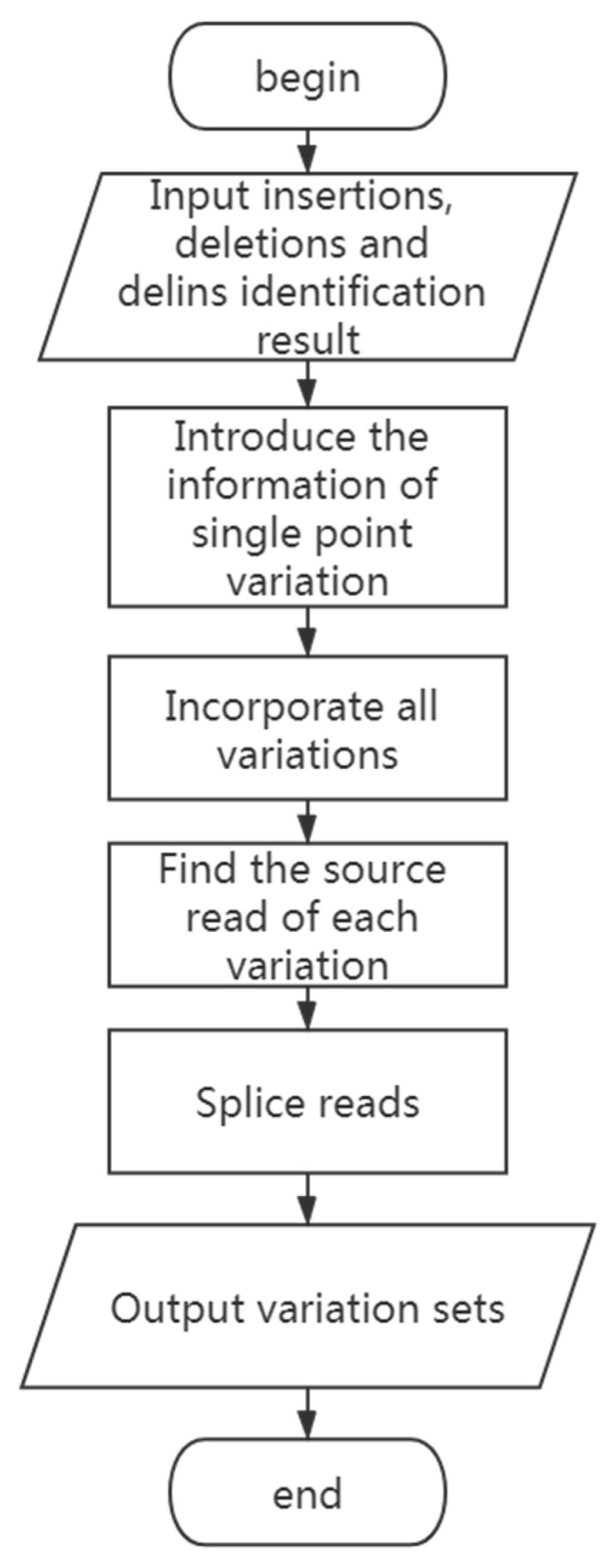
The flow chart of haplotypes estimating process.

The steps of the algorithm are as follows: (1) Introduce the information of variants and incorporate them with insertions, deletions, and delins identification results. (2) Find the source of each variation by walking through the cigar value of each read recorded in the same file. (3) Sequentially splice the reads containing the same variations to obtain a splicing sequence as long as possible. (4) Divide the variations into different sets according to the sequence obtained by splicing. (5) Perform secondary splicing based on the correlation strength between contigs. The program flow chart is shown in Figure 6.

### 2.6. Experimental Methods

The performance of the proposed algorithm was validated with artificial data sets and an independent validation data sets. Since the SVseq3 algorithm can be used for PacBio sequencing data and can detect delins and the position information of the inserted segment, we compared the delInsCaller and SVseq3 under different sequencing errors. In the experiments, precision, recall, and f-measure were used to measure the performance of the algorithm. What’s more, we compared it with the state-of-the-art haplotype estimation tool, HapCUT2. To measure the experimental performances, we selected two widely used haplotype estimating metrics, haplotype accuracy (including switch and mismatch rates), and N50, where switch rate means switch errors as a fraction of possible positions for switch errors, mismatch rate means mismatch errors as a fraction of possible positions for mismatch errors, and N50 means the N50 metric of haplotype completeness.

#### 2.6.1. Simulation Data Sets Experiments

To generate the simulation data sets, a 10 Mbp region was randomly sampled from chromosome 1 of the human reference genome hg19. Then we randomly selected the variation sites on the reference, delete and insert approximately equal-length fragments to simulate the delins variations, and obtain the variant DNA sequence. In the process of simulating the delins, we randomly chose the length of the deletion and the position of the insertion on the reference. To simulate the reads, we used the commonly used simulation tool for the third-generation sequencing data, the PBsim simulator, which can simulate the sequencing process on a DNA sequence file (.fasta) to obtain the read file (.fastq). PBsim simulator can specify the sequencing depth, the mean, and the variance of the read length, the sequencing error rate, and other data characteristics.

#### 2.6.2. Independent Validation Data Sets Experiments

To further verify the results and prove the advantages of the method, we conducted independent validation data sets experiments. We used the sample-fastq function of PBsim to sample data from the real subreads data sets of the NA12878 individual (HG001). The real data were downloaded from GIAB (https://ftp-trace.ncbi.nlm.nih.gov/giab/ftp/data/, accessed on 28 January 2022). Here, we designed two groups of experiments. First, we generated independent validation data sets and performed experiments at different sequencing depths. We set the read length as N (15,000, 1500), the sequencing error rate as 15%, and varied the sequencing depth from 5× to 25×. Again, haplotype estimating metrics, haplotype accuracy (including switch and mismatch rates), and N50 were selected and compared to the state-of-the-art haplotype estimation tool, HapCUT2. Then we compared the precision, recall, and f-measure of delInsCaller on the previous validation data sets and the independent validation data sets, considering that there are too many combinations of read lengths, sequencing depth, delins length, etc. Here, we set the length of delins as 500 bp, the length of read as N (20,000, 2300), the sequencing error rate as 15%, the sequencing depth as 25×, and the number of delins as 100, as an example to generate independent validation data sets.

## 3. Results and Discussion

### 3.1. Experiments under Different Data Characteristics

#### 3.1.1. Experimental Results under Different Sequencing Depths

**Figure 7:**
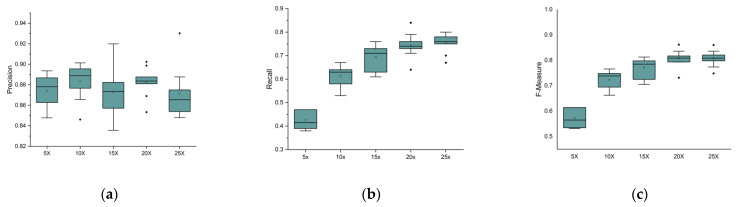
Precision, recall, and f-measure under different sequencing depths: (**a**) description of precision value; (**b**) description of recall value; (**c**) description of the f-measure value. The horizontal lines above and below the boxes represent the maximum and minimum values of the data, respectively. The discrete black dots represent outliers.

We varied the sequencing depth from 5× to 25× and set the read length as N (20,000, 2300), the sequencing error rate as 15%, the length of delins as 500 bp, and the number of delins as 100. For each value of the sequencing depth, we performed ten repeated experiments and drew box diagrams, as shown in Figure 7. From Figure 7, we can conclude that with the increase in the sequencing depth, the recall and f-measure also rise. The reason for this may be that at lower sequencing depth, the reads may not contain enough delins variation signals. However, as the depth deepens, more and more variation signals can be collected.

#### 3.1.2. Experimental Results under Different Lengths of Delins

**Figure 8:**
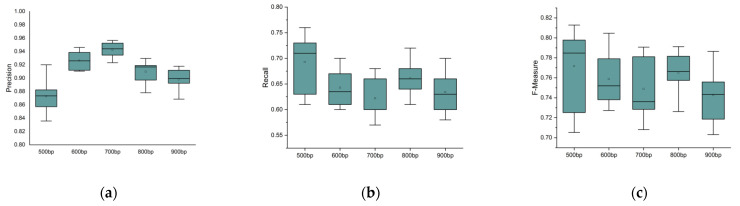
Precision, recall, and f-measure under different lengths of delins: (**a**) description of precision value; (**b**) description of recall value; (**c**) description of the f-measure value. The horizontal lines above and below the boxes represent the maximum and minimum values of the data, respectively. The discrete black dots represent outliers.

We altered the length of delins from 500 bp to1000 bp and set the read length as N (20,000, 2300), the sequencing error rate as 15%, the sequencing depth as 15×, and the number of delins as 100. For each value of the delins length, we performed ten repeated experiments and drew box diagrams, as shown in Figure 8. From Figure 8, we can see that the precision, recall, and f-measure of the delins detection remain almost unchanged as the length of the delins increases. It indicates that the length of delins has little influence on the precision, recall, and f-measure.

### 3.2. Haplotype Phasing Experiments

**Figure 9:**
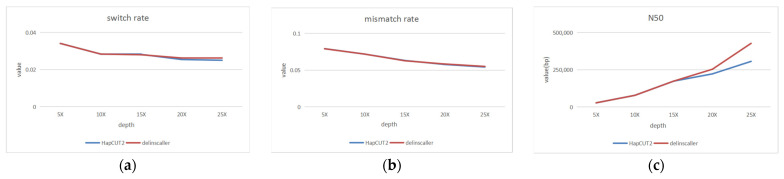
Haplotype estimating under different sequencing depths; (**a**) switch rate; (**b**) mismatch rate; (**c**) N50. The blue line represents the HapCUT2, and the red line represents the dellinsCaller.

In order to test the performance of haplotype estimation, we performed experiments under different sequencing depths and set the read length as N (15,000, 1500) and the sequencing error rate as 15%. We varied the sequencing depth from 5× to 25×. Then a series of experiments were performed for the sequencing data at each depth, and the experimental results are shown in Figure 9. From Figure 9, we can see that at different sequencing depths, our proposed algorithm can be able to improve the N50 values with almost no loss of haplotype accuracy. Moreover, as the depth increases, the advantages become more and more obvious.

### 3.3. Comparison Experiment with SVseq3

**Figure 10:**
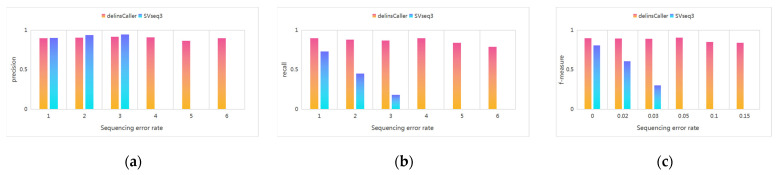
Comparison of delInsCaller and SVseq3 under different sequencing errors: (**a**) precision value; (**b**) recall value; (**c**) f-measure value.

We set the sequencing errors in the experiment as 15%, 10%, 5%, 3%, 2%, and 0%, the read length as N (15,000, 1500), the length of the delins as 500~1000 bp, and the number of delins as 100. The experimental results are shown in Figure 10. As can be seen from the figure, the detection performance of delInsCaller for delins without sequencing errors is similar to that of SVseq3, but delInsCaller is significantly better than SVseq3 if there exist sequencing errors, especially in the case of high sequencing errors. As the sequencing error rate increases, the detection performance of SVseq3 decreases rapidly. However, the increase in sequencing error rate has less effect on delInsCaller, whose f-measure is stable, and the f-measure value of delInsCaller can be reached above 80% in some tests, even when the sequencing error rate reaches 15%. Therefore, the algorithm has a higher tolerance for sequencing errors.

### 3.4. Independent Validation Data Sets Experiments

**Figure 11:**
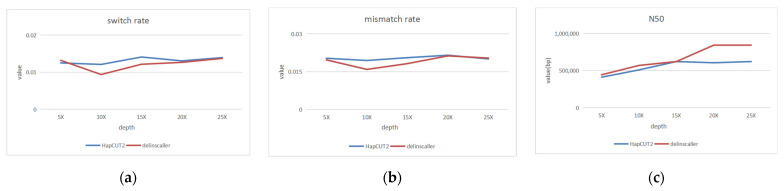
Haplotype estimating under different sequencing depth: (**a**) switch rate; (**b**) mismatch rate; (**c**) N50. The blue line represents the HapCUT2, and the red line represents the dellinsCaller.

First, we set the read length as N (15,000, 1500), the sequencing error rate as 15%, and varied the sequencing depth from 5× to 25×. The experimental results are shown in Figure 11. From Figure 11, we can also see that at different sequencing depths, our proposed algorithm can be able to improve the N50 values with almost no loss of haplotype accuracy. Moreover, as the depth increases, the advantages become more and more obvious. The result is consistent with the previous experimental results on the validation data sets.

**Figure 12:**
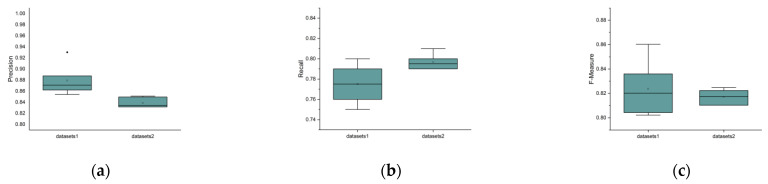
Comparison of detection performance on the previous validation data sets and the independent validation data sets: (**a**) precision value, (**b**) recall value, (**c**) f-measure value. Data set 1 refers to the previous validation data sets, and data set 2 refers to the independent validation data sets. The horizontal lines above and below the boxes represent the maximum and minimum values of the data, respectively. The discrete black dots represent outliers.

We set the length of delins as 500 bp, the length of read as N (20,000, 2300), the sequencing error rate as 15%, the sequencing depth as 25×, and the number of delins as 100 to generate independent validation data sets. Then, we compared the precision, recall, and f-measure of delInsCaller with the previous validation data sets and the independent validation data sets. The experimental results are shown in Figure 12. From Figure 12, we can see that there is little difference in the performance of delInsCaller on the previous validation data sets and the independent validation data sets. In particular, the validation results on both data sets are largely consistent under the f-measure metric.

## 4. Conclusions

In this paper, we focus on identifying delins on haplotype resolution from the PacBio CLR sequencing data. Recent studies emphasized the importance of delins in cancer diagnosis and treatment. Although the CLR reads significantly facilitate delins calling, the existing approaches still encounter computational challenges from the high level of sequencing errors, and often introduce errors in genotyping and phasing delins. So, we proposed an efficient algorithmic pipeline, named delInsCaller, to identify the delins on haplotype resolution from the PacBio CLR sequencing data. delInsCaller has a good tolerance for sequencing errors and can still maintain a high f-measure under a 15% sequencing error rate. It uses a fault-tolerant method by calculating a variation density score, which helps to locate the candidate mutational regions under a high-level of sequencing errors. And it adopts a base association-based contig splicing method, which facilitates contig splicing in the presence of false-positive interference.. We carried out a set of experiments to prove that delInsCaller has a good performance by changing the delins length, sequencing depth, and other data features on the simulation data set. Moreover, we also conducted comparative experiments with several state-of-the-art approaches, e.g., SVseq3, HapCUT2. It is proved that delInsCaller outperformed the existing algorithms. Specifically, it maintained the advantages at ~15% sequencing errors. Therefore, the proposed algorithm is very effective in identifying the delins on haplotype resolution from the PacBio CLR sequencing data with high sequencing errors.

## Data Availability

This is research on developing a bioinformatics tool for sequencing data analysis. We do not generate sequencing data.
